# Impact of long-acting glucocorticoids on ICU mortality in septic patients with acute respiratory failure: a MIMIC-IV based cohort study

**DOI:** 10.3389/fphar.2025.1663974

**Published:** 2025-08-29

**Authors:** Yanhui Deng, Shaoxiang Wang, Shaohua Zhou, Wan Zhao, Aitian Wang, Jingli Gao

**Affiliations:** ^1^ Department of Intensive Medicine, Kailuan General Hospital, Tangshan, China; ^2^ Hebei North University School of Graduate Studies, Zhangjiakou, China

**Keywords:** sepsis, acute respiratory failure, long-acting glucocorticoids, ICU mortality, MIMIC-IV

## Abstract

**Background:**

Long-acting glucocorticoids are frequently administered in the intensive care unit (ICU); however, their precise effect on ICU mortality in septic patients with acute respiratory failure remains inadequately defined. This study aims to investigate whether the use of long-acting glucocorticoids is associated with a reduced mortality rate in this critically ill population.

**Methods:**

Adult patients meeting the Sepsis-3 criteria and simultaneously experiencing acute respiratory failure were retrospectively identified from version 3.0 of the MIMIC-IV (Medical Information Mart for Intensive Care) database. The primary outcome of interest was ICU mortality, defined as death occurring before discharge from the intensive care unit. To minimize baseline confounding, propensity score matching was performed at a 1:3 ratio using nearest-neighbor matching with a caliper width of 0.2 standard deviations. Time-to-event analyses were conducted using Kaplan-Meier survival curves, with statistical significance evaluated via log-rank testing. Additionally, a multivariable Cox proportional hazards regression model was employed to adjust for illness severity and treatment-related covariates, with further exploratory subgroup analyses performed to assess potential effect modifications.

**Results:**

This study analyzed a cohort of 10,707 patients diagnosed in Septic Patients with Acute Respiratory Failure, among whom 2,298 (21.5%) succumbed to ICU mortality. Patients were stratified into glucocorticoid-exposed and unexposed groups based on documented administration of long-acting glucocorticoids during ICU treatment. The crude mortality rate was higher in the unexposed group compared to the glucocorticoid-exposed cohort, and this association remained statistically significant after adjustment in multivariable Cox regression analyses (HR 1.22, 95% CI 1.04–1.43). Subgroup analyses identified significant interaction effects, particularly among patients with malignancies and those receiving continuous renal replacement therapy. Furthermore, propensity score-matched analyses reinforced the primary findings, demonstrating consistent mortality differences between the groups. Sensitivity analysis of different treatment groups showed that the long-acting glucocorticoid group had a significant survival advantage compared to the short-acting glucocorticoid group.

**Conclusion:**

The use of long-acting glucocorticoids was correlated with a reduction in ICU mortality among critically ill by septic patients with acute respiratory failure. This finding indicates a potential survival advantage associated with long-acting glucocorticoid therapy in this high-risk patient population.

## 1 Introduction

Sepsis, defined as life-threatening organ dysfunction caused by a dysregulated host response to infection, remains a leading cause of mortality in intensive care units (ICUs) worldwide (Singer et al., 2016; Venkatesh et al., 2018). It frequently progresses to acute respiratory distress syndrome (ARDS), a severe form of lung injury for which sepsis is the most common etiology (Gorman et al., 2022; ARDS Definition Task Force et al., 2012; Englert et al., 2019; Bellani et al., 2016). The coexistence of sepsis and ARDS is particularly lethal, with mortality rates approaching 40%. Despite advances in supportive care, effective pharmacologic therapies are still lacking (Zhao et al., 2024).

The pathophysiology of sepsis-induced ARDS is driven by an uncontrolled inflammatory cascade. A systemic “cytokine storm,” mediated by tumor necrosis factor-α (TNF-α), interleukin-1β (IL-1β), and interleukin-6 (IL-6), triggers widespread endothelial activation and neutrophil recruitment (Englert et al., 2019; Matthay et al., 2019). This exaggerated inflammatory response disrupts the alveolar–capillary barrier, leading to permeability edema, impaired gas exchange, and the profound hypoxemia characteristic of ARDS (Matthay et al., 2019).

This mechanistic basis provides strong rationale for glucocorticoid therapy, given their potent anti-inflammatory properties mediated through genomic pathways (Timmermans et al., 2019). Upon binding to cytosolic glucocorticoid receptors (GRs), the glucocorticoid–GR complex translocates to the nucleus, modulating gene expression (Barnes, 1998). Key mechanisms include transrepression, in which activated GRs inhibit proinflammatory transcription factors such as nuclear factor-κB (NF-κB), thereby suppressing cytokine production, and transactivation, which induces anti-inflammatory proteins (Rhen and Cidlowski, 2005) (Baschant and Tuckermann, 2010; Coutinho and Chapman, 2011; Vandewalle et al., 2018). Both processes contribute to inflammation resolution, underscoring the complexity of selecting optimal glucocorticoid agents for clinical use (Kleiman and Tuckermann, 2007).

Nevertheless, the clinical efficacy of glucocorticoids in sepsis has remained controversial (Annane et al., 2015; Lamontagne et al., 2018; Rygård et al., 2018). This debate intensified in 2018 following two landmark randomized controlled trials with conflicting outcomes. The ADRENAL trial reported no reduction in 90-day mortality with hydrocortisone in septic shock, whereas the APROCCHSS trial found a significant mortality benefit with hydrocortisone plus fludrocortisone (Annane et al., 2018). Despite these discrepancies in mortality, both trials demonstrated accelerated shock resolution, suggesting that glucocorticoids primarily mitigate organ dysfunction rather than consistently improving survival across heterogeneous septic shock populations.

The therapeutic perspective shifted when attention moved from septic shock to ARDS. The DEXA-ARDS trial, conducted before the COVID-19 pandemic, showed that the long-acting glucocorticoid dexamethasone significantly reduced 60-day mortality in patients with moderate-to-severe ARDS (Villar et al., 2020). This was later reinforced by the RECOVERY trial during the COVID-19 pandemic, which established that dexamethasone reduced 28-day mortality in patients requiring respiratory support (RECOVERY Collaborative Group et al., 2021). Collectively, these findings suggest that glucocorticoids may be most effective in patients with established, inflammation-driven lung injury (Tomazini et al., 2020).

The demonstrated benefit of dexamethasone has reignited debate regarding the optimal glucocorticoid agent (Zhao et al., 2024). Historically, shorter-acting agents such as hydrocortisone have been studied in sepsis, but the mortality reduction observed with long-acting dexamethasone in ARDS has shifted clinical perspectives (Venkatesh et al., 2018; Annane et al., 2018; Villar et al., 2020). Retrospective analyses comparing different glucocorticoids have yielded inconsistent results, leaving considerable uncertainty in managing non-viral sepsis–associated ARDS (Wagner et al., 2021). A critical knowledge gap remains: whether the benefits of long-acting glucocorticoids extend to the broader population of critically ill patients with ARDS secondary to non-viral sepsis.

To address this gap, we conducted a study using the MIMIC-IV database to evaluate the effect of long-acting glucocorticoids on ICU mortality among patients with sepsis and acute respiratory failure, with the goal of informing individualized glucocorticoid treatment strategies in clinical practice.

## 2 Materials and methods

### 2.1 Data source

The data for this retrospective study were obtained from the MIMIC-IV database, version 3.0. Released in July 2024 as an update to MIMIC-IV 2.2, this version contains comprehensive medical records of over 90,000 ICU patients from Beth Israel Deaconess Medical Center (BIDMC) in Boston, Massachusetts, covering the period from 2008 to 2022 (Johnson et al., 2023; MIMIC, 2024). The author of this paper was authorized to access the database (Certificate Number: 61170172) and used it for data extraction.

### 2.2 Study population

The study included patients who met the Sepsis 3.0 criteria, as defined by the Third International Consensus Definitions for Sepsis and Septic Shock (Sepsis-3), which requires suspected or confirmed infection along with an acute increase of at least 2 points in the Sequential Organ Failure Assessment (SOFA) score (Freund et al., 2017; Raith et al., 2017). Patients with acute respiratory failure (ARF) were identified using ICD-9 and ICD-10 codes from the MIMIC-IV database, specifically ICD-9 codes “51,851” and “51,881” and ICD-10 codes “J9600,” “J95821,” “J9601,” and “J9602.” The exclusion criteria were as follows: (1) Age <18 years (2) Non-initial hospital admissions (3) Hospital stay of less than 24 h (4) Abnormal data.

### 2.3 Data extraction

Data were extracted from the MIMIC-IV database (version 3.0) using Structured Query Language (SQL) via Navicat Premium (version 16.3.2). The dataset included demographic variables (age, gender, and BMI), baseline laboratory parameters (white blood cell count, partial pressure of carbon dioxide, partial pressure of oxygen, lactate, total bilirubin, aspartate aminotransferase, blood urea nitrogen, and creatinine), clinical scoring systems [Sequential Organ Failure Assessment (SOFA), Simplified Acute Physiology Score II (SAPS II), and Acute Physiology Score III (APS III)] recorded at the time of ICU admission, vital signs (heart rate, respiratory rate, body temperature, and blood pressure) recorded at the time of ICU admission, comorbidities (malignant cancer and liver disease), use of vasopressin and continuous renal replacement therapy (CRRT), and ICU length of stay.

### 2.4 Exposure and outcomes

Patients were stratified into two groups based on their glucocorticoid regimen: long-acting versus non–long-acting glucocorticoids. Long-acting glucocorticoids, including dexamethasone and betamethasone, were defined by their extended biological half-lives and strong anti-inflammatory properties. The primary outcome was ICU mortality.

### 2.5 Statistical analysis

The statistical analysis consisted of six components. First, baseline characteristics were analyzed as follows: continuous variables were expressed as the mean ± standard deviation for normally distributed data or as the median (interquartile range) for non-normally distributed data, while categorical variables were reported as frequencies and percentages. Group differences in means and proportions were assessed using the Welch two-sample t-test, the Wilcoxon rank-sum test, and the chi-square test, as appropriate.

Second, propensity score matching (PSM) was applied to adjust for baseline differences in the likelihood of glucocorticoid administration. Patients in the glucocorticoid group were defined as those who received the medication throughout their ICU stay. A 1:3 matching ratio was used to pair patients in the treatment group with untreated controls. The standardized mean difference (SMD) was calculated before and after matching to evaluate the effectiveness of PSM in balancing pretreatment covariates between groups. Third, unadjusted survival curves were constructed using the Kaplan-Meier (KM) method, and differences between groups were assessed using the log-rank test. Fourth, Cox regression analysis was performed to examine the association between glucocorticoid use and ICU mortality. A multivariate Cox regression model was further employed to adjust for potential confounding factors. Fifth, subgroup analyses were conducted to explore variations in the effects of glucocorticoids across different patient subgroups, stratified by age, gender, comorbidities, and illness severity scores. Sixth, sensitivity analysis is used to determine the impact of different medication groups on the risk of death within the ICU.

A P-value of <0.05 was considered statistically significant. All statistical analyses were performed using R software (version 4.2.2; R Foundation for Statistical Computing, Vienna, Austria).

## 3 Results

### 3.1 Baseline characteristics and clinical outcomes of the study participants

**FIGURE 1:**
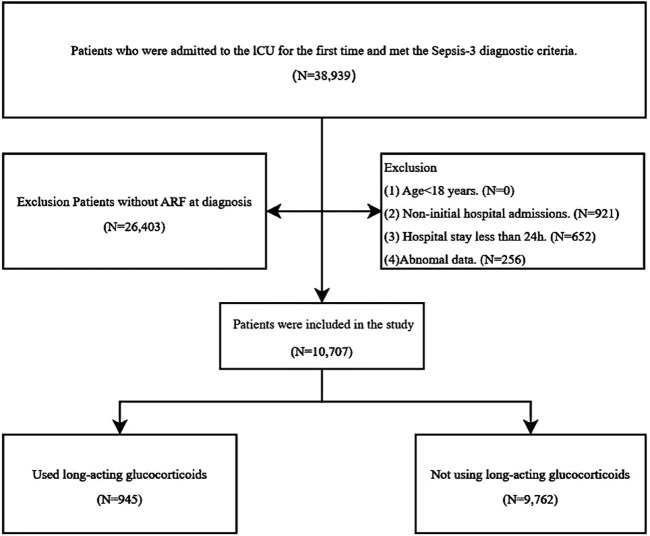
The flowchart illustrating the inclusion and exclusion process.

Figure 1 illustrates the flowchart detailing the selection process for the study population. After applying the inclusion and exclusion criteria, a total of 10,707 patients were enrolled in the study, among whom 945 (8.8%) received long-acting glucocorticoids (Table 1). The median age of the study cohort was 68 years, with glucocorticoid users being significantly younger than non-users (64 vs. 69 years; p < 0.001). A notable difference was observed in sex distribution, with a higher proportion of males (57.1%) than females (42.9%; p = 0.006). The median BMI was 29, slightly lower in the glucocorticoid group compared to non-users (28 vs. 29; p = 0.002).

**TABLE 1:** Baseline characteristics.

Variable	Long-acting glucocorticoids			p-value
	Overall, N = 10,707	Use, N = 945	No-use, N = 9,762	
Age (y)	68 (57, 79)	64 (54, 74)	69 (57, 79)	<0.001
Gender (%)				0.006
Male	6,110 (57.1%)	499 (52.8%)	5,611 (57.5%)	
Female	4,597 (42.9%)	446 (47.2%)	4,151 (42.5%)	
BMI	29 (25, 33)	28 (25, 32)	29 (25, 33)	0.002
SOFA	7.0 (4.0, 10.0)	6.0 (3.0, 8.0)	7.0 (4.0, 10.0)	<0.001
APSIII	53 (40, 70)	49 (36, 66)	53 (40, 70)	<0.001
SAPSII	42 (34, 53)	41 (32, 52)	43 (34, 53)	0.001
HR (bpm)	92 (78, 107)	93 (78, 110)	91 (78, 107)	0.055
SBP (mmHg)	119 (103, 137)	122 (105, 140)	119 (103, 136)	<0.001
DBP(mmHg)	67 (56, 79)	69 (59, 83)	67 (56, 79)	<0.001
RR (insp/min)	20 (16, 24)	20 (16, 25)	20 (16, 24)	0.056
Temperature	36.83 (36.50, 37.22)	36.89 (36.50, 37.28)	36.83 (36.50, 37.22)	0.025
WBC (K/uL)	12 (8, 17)	11 (7, 16)	12 (8, 17)	<0.001
PCO2 (mmHg)	42 (36, 50)	40 (34, 47)	42 (36, 50)	<0.001
PO2 (mmHg)	87 (54, 152)	93 (54, 158)	87 (53, 151)	0.112
Lactate (mmol/L)	1.80 (1.21, 2.80)	1.70 (1.20, 2.47)	1.80 (1.24, 2.90)	<0.001
INR	1.30 (1.20, 1.70)	1.20 (1.10, 1.50)	1.30 (1.20, 1.70)	<0.001
Bilirubintotal (mg/dL)	0.70 (0.40, 1.30)	0.60 (0.34, 1.04)	0.70 (0.40, 1.30)	<0.001
AST (IU/L)	45 (26, 100)	39 (24, 74)	46 (27, 103)	<0.001
Ureanitrogen	25 (16, 41)	21 (14, 34)	25 (16, 42)	<0.001
Creatinine (mg/dL)	1.20 (0.80, 1.90)	0.90 (0.70, 1.60)	1.20 (0.80, 2.00)	<0.001
CRRT (%)				0.017
N	9,381 (87.6%)	851 (90.1%)	8,530 (87.4%)	
Y	1,326 (12.4%)	94 (9.9%)	1,232 (12.6%)	
Vasopressin (%)				<0.001
N	8,674 (81.0%)	828 (87.6%)	7,846 (80.4%)	
Y	2,033 (19.0%)	117 (12.4%)	1,916 (19.6%)	
Malignant cancer (%)				<0.001
N	8,993 (84.0%)	710 (75.1%)	8,283 (84.8%)	
Y	1,714 (16.0%)	235 (24.9%)	1,479 (15.2%)	
Liver disease (%)				<0.001
N	9,272 (86.6%)	866 (91.6%)	8,406 (86.1%)	
Y	1,435 (13.4%)	79 (8.4%)	1,356 (13.9%)	
ICU LOS (days)	6 (3, 11)	7 (3, 14)	5 (3, 11)	<0.001
ICU mortality (n (%))				0.005
N	8,409 (78.5%)	776 (82.1%)	7,633 (78.2%)	
Y	2,298 (21.5%)	169 (17.9%)	2,129 (21.8%)	

BMI, body mass index; SOFA, sequential organ failure assessment; APS III, Acute Physiology Score III; SAPS II, Simplified Acute Physiology Score II; HR, heart rate; SBP, systolic blood pressure; DBP, diastolic blood pressure; RR, respiratory rate; WBC, white blood cell count; PCO_2_, partial pressure of carbon dioxide; PO_2_, partial pressure of oxygen; INR, international normalized ratio; AST, aspartate aminotransferase; CRRT, continuous renal replacement therapy; ICU, intensive care unit; LOS, length of stay.

Patients receiving glucocorticoids had lower SOFA scores (6.0 vs. 7.0; p < 0.001), APS III scores (49 vs. 53; p < 0.001), and SAPS II scores (41 vs. 43; p = 0.001), suggesting a potentially lower severity of illness. In terms of hemodynamic parameters, patients in the glucocorticoid group had significantly higher systolic (122 vs. 119 mmHg; p < 0.001) and diastolic blood pressure (69 vs. 67 mmHg; p < 0.001) than non-users. The median body temperature was slightly elevated in the glucocorticoid group (36.89 °C vs. 36.83 °C; p = 0.025).

Regarding laboratory findings, patients receiving glucocorticoids had significantly lower median WBC counts (11 vs. 12 ×10^9^/L; p < 0.001) and INR values (1.2 vs. 1.3; p < 0.001) but exhibited higher total bilirubin levels (0.60 vs. 0.70 mg/dL; p < 0.001), AST (39 vs. 46 IU/L; p < 0.001), blood urea nitrogen (21 vs. 25 mg/dL; p < 0.001), and creatinine levels (0.90 vs. 1.20 mg/dL; p < 0.001). Additionally, lactate levels were significantly lower among glucocorticoid users (1.70 vs. 1.80 mmol/L; p < 0.001).

Comorbidity analysis revealed significant differences in the prevalence of malignant cancer (24.9% vs. 15.2%; p < 0.001) and liver disease (8.4% vs. 13.9%; p < 0.001) between glucocorticoid users and non-users. Furthermore, patients in the glucocorticoid group had a longer ICU stay (7 vs. 5 days; p < 0.001). The in-ICU mortality rate was lower among glucocorticoid users compared to non-users (17.9% vs. 21.8%; p = 0.005).

### 3.2 Baseline characteristics and clinical outcomes following propensity score matching

To mitigate the influence of confounding bias, we conducted 1:3 propensity score matching (PSM) with a caliper width of 0.2, stratifying patients based on glucocorticoid administration. This matching process successfully paired 939 patient pairs. Importantly, post-matching analysis demonstrated that baseline characteristics were well balanced between the two groups, with standardized mean differences (SMDs) below 0.2 for all variables (Table 2), indicating a high degree of comparability.

**TABLE 2:** Baseline characteristics of patients before and after propensity score matching.

Variables	Before matching			After matching		
	No-use	Use	SMD^△^	No-use	Use	SMD
N	9,762	945		2,757	939	
Age (yr)	66.99 (16.16)	63.14 (15.60)	−0.247	63.76 (17.42)	63.24 (15.57)	−0.011
Gender-Male (%)	5,611 (57.5)	499 (52.8)	−0.094	1,438 (52.2)	497 (52.9)	0.023
BMI	29.58 (6.78)	29.04 (6.71)	−0.081	29.11 (6.66)	29.06 (6.73)	−0.003
SOFA	7.19 (3.93)	6.08 (3.77)	−0.293	6.15 (3.58)	6.08 (3.78)	−0.009
APSIII	57.35 (23.83)	52.65 (23.22)	−0.202	52.57 (22.43)	52.53 (23.17)	0.003
SAPSII	44.38 (14.99)	42.94 (15.46)	−0.093	42.77 (15.13)	42.86 (15.46)	0.001
HR (bpm)	93.16 (20.63)	94.70 (21.69)	0.071	94.49 (21.00)	94.64 (21.64)	−0.001
SBP (mmHg)	120.33 (24.09)	123.35 (24.09)	0.125	123.27 (24.19)	123.17 (23.98)	−0.013
DBP(mmHg)	68.75 (18.05)	71.80 (17.96)	0.170	71.45 (18.45)	71.63 (17.78)	−0.007
RR (insp/min)	20.89 (6.14)	21.39 (6.56)	0.076	21.43 (6.36)	21.36 (6.53)	−0.016
Temperature	36.87 (0.74)	36.93 (0.80)	0.074	36.91 (0.74)	36.93 (0.80)	0.026
WBC (K/uL)	13.59 (7.68)	12.52 (7.83)	−0.136	12.57 (6.82)	12.54 (7.84)	0.004
PCO2 (mmHg)	44.33 (12.89)	42.05 (11.53)	−0.197	42.19 (11.61)	42.06 (11.54)	−0.001
PO2 (mmHg)	120.06 (95.21)	122.99 (93.89)	0.031	123.84 (91.99)	122.99 (94.06)	−0.010
Lactate (mmol/L)	2.49 (2.09)	2.19 (1.72)	−0.178	2.23 (1.80)	2.19 (1.72)	−0.016
INR	1.59 (0.77)	1.39 (0.52)	−0.389	1.39 (0.48)	1.39 (0.52)	−0.002
Bilirubintotal (mg/dL)	1.63 (3.23)	1.14 (2.27)	−0.217	1.15 (2.09)	1.14 (2.28)	0.002
AST (IU/L)	198.33 (630.84)	117.35 (331.52)	−0.244	118.50 (368.94)	117.80 (332.53)	0.002
Ureanitrogen	32.42 (23.53)	28.15 (22.06)	−0.193	28.57 (21.82)	28.14 (22.06)	−0.012
Creatinine (mg/dL)	1.69 (1.44)	1.41 (1.29)	−0.222	1.42 (1.21)	1.41 (1.29)	−0.002
CRRT (%)	1,232 (12.6)	94 (9.9)	−0.089	266 (9.6)	94 (10.0)	0.015
Vasopressin (%)	1916 (19.6)	117 (12.4)	−0.220	340 (12.3)	117 (12.5)	0.009
Malignant cancer (%)	1,479 (15.2)	235 (24.9)	0.225	656 (23.8)	229 (24.4)	−0.009
Liver disease (%)	1,356 (13.9)	79 (8.4)	−0.200	237 (8.6)	79 (8.4)	−0.003
ICU LOS (days)	5 (3, 11)	7 (3, 14)	0.201	6 (3,11)	7 (3,14)	0.193
In-ICU mortality (n (%))	2,129 (21.8%)	169 (17.9%)	−0.098	508 (18.4%)	168 (17.9%)	0.014

SMD, standardized mean difference.

### 3.3 Survival analysis

**FIGURE 2:**
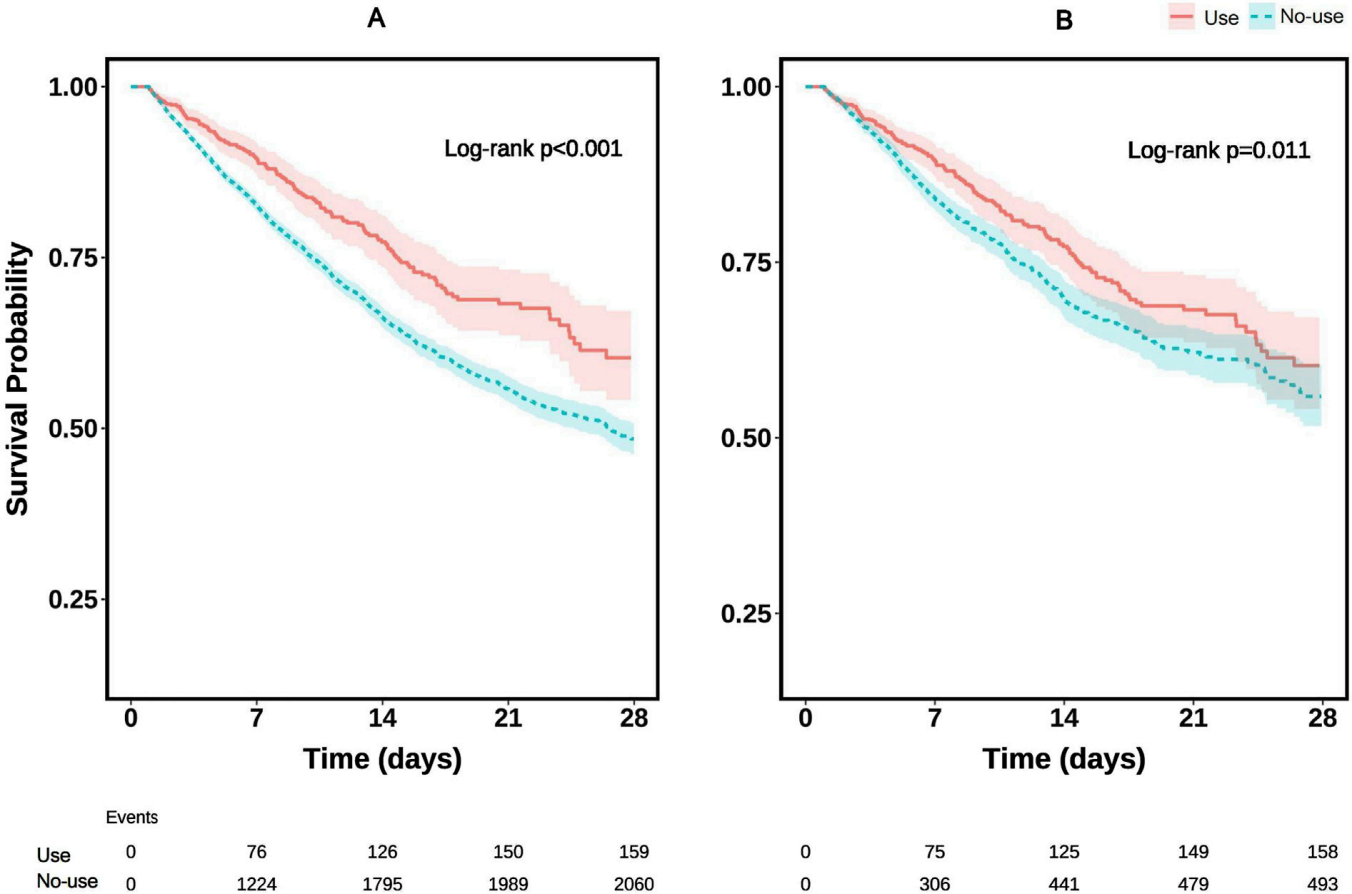
Kaplan–Meier survival curves for ICU mortality: **(A)** before propensity score matching (PSM); **(B)** after PSM.

The Kaplan-Meier survival curve was generated to visually compare mortality risk between the two patient groups (Figure 2A). The results demonstrated that ICU survival rates were higher among sepsis patients with acute respiratory failure who received glucocorticoids compared to those who did not. Notably, this trend persisted even after conducting the same analysis on the propensity score-matched (PSM) dataset (Figure 2B), reinforcing the robustness of the findings. The log-rank test yielded p-values of less than 0.05 across all survival curves, indicating statistically significant differences between the groups.

### 3.4 Cox proportional-hazards regression model assessing the association between glucocorticoids use and the risk of death

We performed a multivariate Cox regression analysis to assess the association between long-acting glucocorticoid use and the primary outcome (Table 3). The analysis was conducted using three models, each progressively adjusting for additional covariates.

**TABLE 3:** Multivariate Cox regression analysis.

Characteristic	Model 1			Model 2			Model 3		
	HR^a^	95% CI^a^	p-value	HR^a^	95% CI^a^	p-value	HR^a^	95% CI^a^	p-value
Original data Long-acting glucocorticoids									
Use	-	-		-	-		-	-	
No-use	1.50	1.28, 1.76	<0.001	1.44	1.23, 1.68	<0.001	1.22	1.04, 1.43	0.015
After PSM Long-acting glucocorticoids									
Use	-	-		-	-		-	-	
No-use	1.26	1.05, 1.50	0.011	1.26	1.05, 1.50	0.010	1.27	1.06, 1.52	0.008

^a^ HR, hazard ratio; CI, confidence interval.

Model 1: no covariates were adjusted.

Model 2: adjusted for Age and Gender.

Model 3: adjusted for Age, Gender, BMI, CRRT, Cancer, Liver disease, SOFA, APSIII, SAPSII, Vasopressin, HR, SBP, DBP, RR, Temperature, WBC, PCO2, PO2, Lactate, INR, Bilirubin total, AST, Urea nitrogen, and Creatinine.

In the unadjusted Model 1, non-use of long-acting glucocorticoids was associated with a significantly higher hazard ratio (HR = 1.50, 95% CI: 1.28–1.76, p < 0.001) compared to glucocorticoid use. After adjusting for age and gender in Model 2, the association remained statistically significant, though the effect size was slightly attenuated (HR = 1.44, 95% CI: 1.23–1.68, p < 0.001). Further adjustments in Model 3, which included additional clinical and biochemical covariates—such as continuous renal replacement therapy (CRRT), malignancy, liver disease, SOFA score, APS III, SAPS II, BMI, vasopressin use, hemodynamic parameters, and laboratory indices—showed that the association persisted, but was further attenuated (HR = 1.22, 95% CI: 1.04–1.43, p = 0.015). In the propensity score-matched (PSM) cohort, the association between non-use of long-acting glucocorticoids and the outcome remained significant across all models: Model 1 (HR = 1.26, 95% CI: 1.05–1.50, p = 0.011), Model 2 (HR = 1.26, 95% CI: 1.05–1.50, p = 0.010), and Model 3 (HR = 1.27, 95% CI: 1.06–1.52, p = 0.008). These results suggest that, even after rigorous adjustment for potential confounders, non-use of long-acting glucocorticoids remains an independent risk factor for the outcome.

### 3.5 Subgroup analysis

**FIGURE 3:**
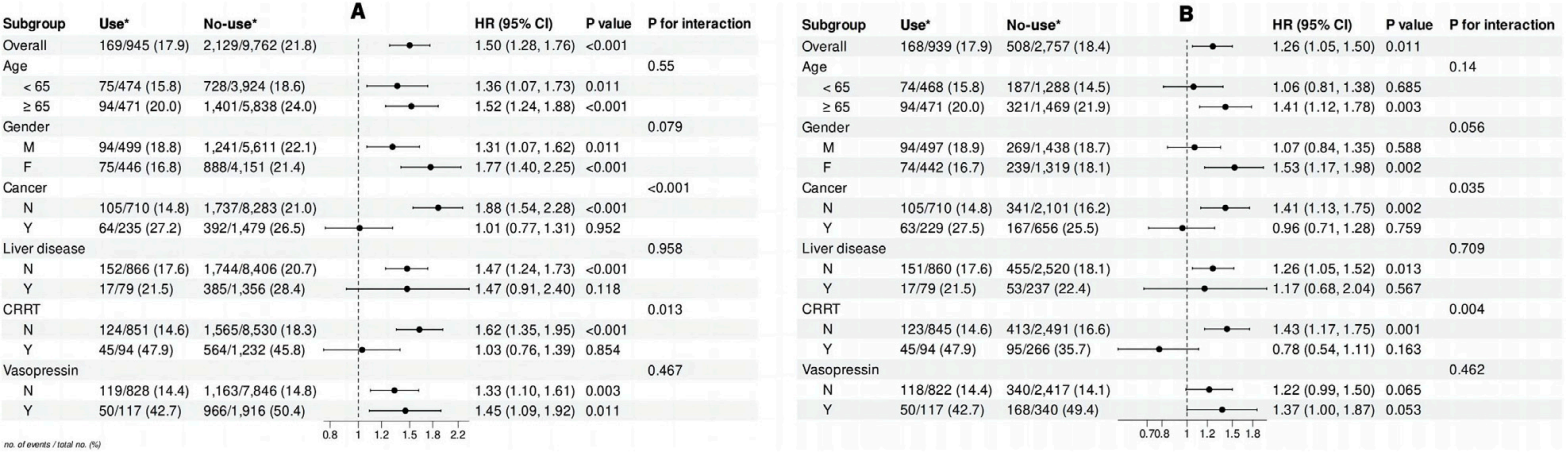
Forest plot illustrating the results of subgroup analyses on the relationship between long-acting glucocorticoid use and ICU mortality: **(A)** before propensity score matching (PSM); **(B)** after PSM.

Subgroup analyses (Figure 3A) revealed that the increased mortality risk associated with non-use of long-acting glucocorticoids was significant in patients aged ≥65 years (HR = 1.41, 95% CI: 1.12–1.78, p = 0.003), but not in those aged <65 years (p for interaction = 0.14). A stronger association was observed in females (HR = 1.53, 95% CI: 1.17–1.98, p = 0.002) compared to males. The effect remained significant in patients without cancer (HR = 1.41, 95% CI: 1.13–1.75, p = 0.002), but not in those with cancer (p for interaction = 0.035). Notably, the association was significant in patients who did not receive continuous renal replacement therapy (CRRT) (HR = 1.43, 95% CI: 1.17–1.75, p = 0.001), but not in those undergoing CRRT (p for interaction = 0.004). No significant interactions were found in other subgroups. The PSM results reinforced the robustness of our findings (Figure 3B), particularly in older patients, females, and those without cancer, while highlighting a notable difference in the CRRT subgroup.

### 3.6 Sensitivity analysis

To further explore the heterogeneity of treatment effects across glucocorticoid regimens, a sensitivity analysis was performed. The cohort was stratified into four subgroups: Use of Long-acting glucocorticoids only, Use of short-acting glucocorticoids only, Use of both short- and long-acting glucocorticoids, and Without short- or long-acting glucocorticoid use. Baseline characteristics differed substantially among groups, with patients in the short-acting–only group exhibiting greater illness severity and more frequent organ support compared with those in the long-acting–only (Supplementary Table S1). Across three Cox models (unadjusted; adjusted for age and sex; and fully adjusted for demographics, comorbidities, organ support, and key physiologic/laboratory covariates), exclusive use of long-acting glucocorticoids was associated with the most favorable outcomes (Supplementary Table S2). In the fully adjusted model, the risk of ICU death was significantly higher in the no-glucocorticoid group (HR, 1.24; 95% CI, 1.03–1.50; P = 0.024) and the short-acting–only group (HR, 1.56; 95% CI, 1.26–1.92; P < 0.001), whereas the combined-use group showed a nonsignificant association (HR, 1.22; 95% CI, 0.88–1.71; P = 0.238). Kaplan–Meier survival curves were consistent with these results, demonstrating the most favorable survival in the long-acting–only group and the least favorable in the short-acting–only group (Supplementary Figure S1). Taken together, these sensitivity analyses—adjusted for measured confounders and supported by both regression models and survival-curve findings—confirm that exclusive long-acting glucocorticoid therapy is associated with lower ICU mortality compared with other exposure patterns in this cohort.

## 4 Discussion

This study demonstrates that the use of long-acting glucocorticoids is independently associated with reduced ICU mortality among critically ill patients with sepsis and acute respiratory failure. This association persisted after extensive adjustment for confounding factors—including illness severity scores, comorbidities, and therapeutic interventions—and was further supported by propensity score–matched analyses. The observed survival benefit is consistent with emerging evidence highlighting the immunomodulatory advantages of long-acting glucocorticoids in hyperinflammatory states. Moreover, our findings provide novel insights into their differential effects across clinically relevant subgroups. Sensitivity analyses further confirmed that, compared with short-acting glucocorticoids, long-acting agents continued to confer significant survival benefits. Collectively, these results suggest that, within the pathophysiologic setting of inflammation-driven pulmonary injury, the selection of a potent, long-half-life glucocorticoid may yield greater clinical benefit than shorter-acting regimens.

Our findings can be interpreted in the context of previous clinical trials evaluating corticosteroids in sepsis and ARDS. In the CORTICUS trial, low‐dose hydrocortisone (200 mg/day) accelerated shock reversal but did not improve 28‐days survival (34% vs. 32% mortality, P = 0.51) (Sprung et al., 2008). Both studies demonstrated faster shock resolution and reduced duration of initial mechanical ventilation in the steroid-treated groups, but neither observed an overall mortality benefit. In contrast, the APROCCHSS trial (n = 1,241), which evaluated combination therapy with hydrocortisone (50 mg IV every 6 h) plus fludrocortisone (50 μg daily), reported improved survival. Ninety‐day mortality was 43% in the steroid-fludrocortisone group versus 49% in placebo (relative risk, 0.88; 95% CI, 0.78–0.99; P = 0.03) (Annane et al., 2018). A recent network meta-analysis encompassing over 95,000 patients similarly concluded that hydrocortisone combined with fludrocortisone reduced short-term mortality compared with placebo (OR ≈ 0.79; NNT ≈21) (Lai et al., 2024). Collectively, these findings suggest that mineralocorticoid supplementation may play a critical role in septic shock, whereas hydrocortisone alone provides limited survival benefit. Notably, neither CORTICUS nor ADRENAL directly compared dexamethasone with hydrocortisone, highlighting that our study contributes novel evidence regarding the choice of corticosteroid type.

In contrast to trials in septic shock, several recent RCTs have specifically examined ARDS arising from pulmonary sepsis, in which dexamethasone has demonstrated clear benefits. In the 2020 DEXA-ARDS trial involving patients with moderate–severe ARDS, early administration of dexamethasone (20 mg IV daily) significantly reduced 60‐days mortality (21% vs. 36%) and increased ventilator-free days by approximately 5 days (mean difference, +4.8 days; P < 0.0001) (Villar et al., 2020). Similarly, the RECOVERY trial in COVID-19–related ARDS showed that dexamethasone 6 mg daily reduced 28‐days mortality by approximately 17% overall, with the greatest effect observed in mechanically ventilated patients (29.3% vs. 41.4%; rate ratio, 0.64; 95% CI, 0.51–0.81) (RECOVERY Collaborative Group et al., 2021). These findings support a mortality benefit of dexamethasone in acute lung injury. Collectively, the RCT evidence suggests that long‐acting glucocorticoids can be advantageous in ARDS, whereas in shock without concurrent mineralocorticoid supplementation, survival benefit remains uncertain. Our results—demonstrating improved ICU survival with long‐acting steroids in septic respiratory failure—are consistent with the ARDS trials (DEXA-ARDS, RECOVERY) and with the APROCCHSS findings, interpreted as evidence that higher steroid potency or effective mineralocorticoid activity can enhance outcomes (Villar et al., 2020). Notably, current clinical guidelines reflect this ambiguity: the Surviving Sepsis Campaign provides only a weak recommendation for IV corticosteroid use in vasopressor-dependent shock, acknowledging the mixed results from prior trials (Zhu et al., 2025).

The pathophysiology of sepsis-induced ARDS provides a strong mechanistic basis for our findings. The dysregulated host immune response triggers a “cytokine storm” that damages the alveolar-capillary barrier, resulting in non-cardiogenic pulmonary edema (Son et al., 2021; Nedeva, 2021; Messina et al., 2021). Glucocorticoids exert potent anti-inflammatory effects by binding to the glucocorticoid receptor (GR) and modulating gene expression, primarily via inhibition of pro-inflammatory transcription factors such as NF-κB (Rhen and Cidlowski, 2005; Balkrishna et al., 2023; Li and Cummins, 2022). In both ARDS and septic shock, excessive inflammation drives capillary leak and organ dysfunction; steroids can attenuate this pulmonary injury and systemic response (Al-Yousif et al., 2024). The distinct pharmacological properties of long-acting glucocorticoids, such as dexamethasone, provide a plausible explanation for their superior efficacy in this setting. Dexamethasone exhibits approximately 20–30 times the anti-inflammatory potency of hydrocortisone, demonstrates higher GR binding affinity, and has a prolonged biological half-life (36–54 h), ensuring sustained suppression of the continuous inflammatory cascade characteristic of ARDS (Cho and Suh, 2024; Luyt et al., 2020; Zhang et al., 2023). In contrast, the short half-life of hydrocortisone may result in fluctuating GR activation and potential rebound inflammation. Moreover, dexamethasone’s lack of mineralocorticoid activity mitigates the risk of sodium and water retention, a critical consideration in ARDS, where positive fluid balance is associated with poor outcomes (Cho and Suh, 2024; Zhang et al., 2023). Taken together, these pharmacological advantages are mechanistically well aligned with the pathophysiology of sepsis-induced ARDS.

Our subgroup analyses offer additional insights into heterogeneity of treatment effects. The protective impact of long-acting glucocorticoids was more pronounced in patients aged ≥65 years, potentially reflecting age-related alterations in the hypothalamic-pituitary-adrenal axis (Veldhuis et al., 2005). In contrast, the benefit was attenuated among patients with malignancies, likely due to complex interactions with baseline immunosuppression from the underlying cancer or its therapies (Zhang et al., 2009). A novel observation was the significant interaction with continuous renal replacement therapy (CRRT); the protective effect was evident only in patients not receiving CRRT. This may be attributable to pharmacokinetic factors, as dexamethasone’s high protein binding and large volume of distribution render it less susceptible to clearance by CRRT, maintaining more stable drug concentrations (Brochard et al., 2010). These findings must be weighed against potential risks. Historically, concerns regarding adverse effects such as secondary infections have limited glucocorticoid use (Hsu et al., 2024; Venet and Monneret, 2018). However, recent large-scale meta-analyses suggest that contemporary, guideline-recommended dosing does not substantially increase the risk of secondary infection or gastrointestinal bleeding in critically ill patients, although it is associated with manageable hyperglycemia and hypernatremia (Windsor et al., 1993). Considering the potential for meaningful mortality reduction, the overall risk-benefit profile appears favorable in this population.

The strengths of our study include its large, real-world cohort and the application of robust statistical methodologies. Nevertheless, the retrospective design imposes inherent limitations. Unmeasured confounders—such as specific pathogens, detailed ventilator parameters, or precise information regarding the timing and dosage of glucocorticoid administration—may remain despite extensive adjustment. Additionally, the absence of standardized treatment protocols introduces therapeutic heterogeneity, and the single-center nature of the dataset may limit generalizability. Consequently, although our study offers strong, hypothesis-generating evidence, it cannot establish causality. A definitive, multicenter randomized controlled trial directly comparing long-acting and short-acting glucocorticoids in patients with sepsis-induced acute respiratory failure is urgently needed to provide the highest level of evidence.

## 5 Conclusion

In this large-scale retrospective analysis, the administration of long-acting glucocorticoids was associated with reduced ICU mortality in septic patients with acute respiratory failure. Our findings suggest that the sustained anti-inflammatory and immunomodulatory properties of long-acting agents may confer unique advantages in sepsis-associated ARF. However, prospective randomized trials are warranted to validate these observations and further elucidate the optimal glucocorticoid strategy in this high-risk population.

## Data Availability

The raw data supporting the conclusions of this article will be made available by the authors, without undue reservation.

## Supplementary material

SUPPLEMENTARY FIGURE S1 Kaplan–Meier survival curves for four subgroups: Use of Long-acting glucocorticoids only, Use of short-acting glucocorticoids only, Use of both short- and long-acting glucocorticoids, and Without short- or long-acting glucocorticoid use.
