# Supplementary material for: Impact of long-acting glucocorticoids on ICU mortality in septic patients with acute respiratory failure: a MIMIC-IV based cohort study
Source: Front Pharmacol. 2025 Aug 29;16:1663974. doi: 10.3389/fphar.2025.1663974 (PMC12426184; doi:10.3389/fphar.2025.1663974)
Supplement: Supplementary file 1 [file Table1.docx]

**Supplementary Table S1.** Baseline of different glucocorticoid treatment groups

| **Characteristic** | **Glucocorticoid treatment group** | | | | | **p-value** |
| --- | --- | --- | --- | --- | --- | --- |
|  | **Overall  N = 10,707** | **Use of Long-acting glucocorticoids only  N = 783** | **Without short- or long-acting glucocorticoid use  N = 8,559** | **Use of both short- and long-acting glucocorticoids  N = 162** | **Use of short-acting glucocorticoids only  N = 1,203** |  |
| Age (y) | 68 (57, 79) | 65 (55, 75) | 69 (57, 80) | 64 (54, 72) | 67 (57, 76) | <0.001 |
| Gender (%) |  |  |  |  |  | 0.023 |
| Male | 6,110 (57.1%) | 421 (53.8%) | 4,924 (57.5%) | 78 (48.1%) | 687 (57.1%) |  |
| Female | 4,597 (42.9%) | 362 (46.2%) | 3,635 (42.5%) | 84 (51.9%) | 516 (42.9%) |  |
| BMI | 29 (25, 33) | 28 (25, 32) | 29 (25, 33) | 27 (25, 32) | 29 (25, 33) | 0.020 |
| SOFA | 7.0 (4.0, 10.0) | 5.0 (3.0, 8.0) | 6.0 (4.0, 9.0) | 7.5 (5.0, 11.0) | 9.0 (6.0, 12.0) | <0.001 |
| APSIII | 53 (40, 70) | 46 (34, 62) | 52 (39, 68) | 65 (51, 79) | 68 (50, 88) | <0.001 |
| SAPSII | 42 (34, 53) | 40 (31, 50) | 42 (33, 52) | 49 (38, 60) | 50 (40, 62) | <0.001 |
| HR (bpm) | 92 (78, 107) | 91 (77, 107) | 91 (78, 106) | 102 (89, 119) | 97 (82, 112) | <0.001 |
| SBP (mmHg) | 119 (103, 137) | 125 (107, 142) | 120 (104, 137) | 115 (100, 131) | 111 (96, 127) | <0.001 |
| DBP (mmHg) | 67 (56, 79) | 70 (60, 84) | 67 (56, 79) | 66 (56, 80) | 63 (54, 75) | <0.001 |
| RR (insp/min) | 20 (16, 24) | 20 (16, 25) | 20 (16, 24) | 22 (18, 27) | 21 (17, 25) | <0.001 |
| Temperature | 36.83 (36.50, 37.22) | 36.89 (36.56, 37.33) | 36.83 (36.50, 37.22) | 36.78 (36.50, 37.28) | 36.78 (36.44, 37.11) | <0.001 |
| WBC (K/uL) | 12 (8, 17) | 11 (8, 16) | 12 (8, 17) | 10 (6, 17) | 12 (8, 19) | <0.001 |
| PCO2 （mmHg） | 42 (36, 50) | 40 (34, 47) | 42 (36, 50) | 41 (33, 48) | 41 (35, 50) | <0.001 |
| PO2 （mmHg） | 87 (54, 152) | 96 (57, 168) | 88 (55, 154) | 80 (43, 121) | 74 (47, 130) | <0.001 |
| Lactate (mmol/L) | 1.80 (1.21, 2.80) | 1.60 (1.20, 2.40) | 1.70 (1.20, 2.80) | 1.90 (1.40, 3.10) | 2.30 (1.50, 4.00) | <0.001 |
| INR | 1.30 (1.20, 1.70) | 1.20 (1.10, 1.40) | 1.30 (1.20, 1.67) | 1.40 (1.20, 1.60) | 1.50 (1.20, 2.00) | <0.001 |
| Bilirubintotal (mg/dL) | 0.70 (0.40, 1.30) | 0.50 (0.30, 0.92) | 0.68 (0.40, 1.20) | 0.80 (0.40, 1.70) | 0.80 (0.46, 2.30) | <0.001 |
| AST (IU/L) | 45 (26, 101) | 37 (23, 70) | 45 (27, 98) | 48 (29, 130) | 53 (27, 140) | <0.001 |
| Ureanitrogen | 25 (16, 41) | 20 (13, 32) | 24 (16, 41) | 29 (17, 46) | 30 (19, 51) | <0.001 |
| Creatinine (mg/dL) | 1.20 (0.80, 1.90) | 0.90 (0.70, 1.50) | 1.20 (0.80, 1.90) | 1.10 (0.80, 2.30) | 1.60 (1.00, 2.70) | <0.001 |
| CRRT (%) |  |  |  |  |  | <0.001 |
| N | 9,381 (87.6%) | 727 (92.8%) | 7,728 (90.3%) | 124 (76.5%) | 802 (66.7%) |  |
| Y | 1,326 (12.4%) | 56 (7.2%) | 831 (9.7%) | 38 (23.5%) | 401 (33.3%) |  |
| Vasopressin (%) |  |  |  |  |  | <0.001 |
| N | 8,674 (81.0%) | 707 (90.3%) | 7,186 (84.0%) | 121 (74.7%) | 660 (54.9%) |  |
| Y | 2,033 (19.0%) | 76 (9.7%) | 1,373 (16.0%) | 41 (25.3%) | 543 (45.1%) |  |
| Malignant cancer (%) |  |  |  |  |  | <0.001 |
| N | 8,993 (84.0%) | 585 (74.7%) | 7,289 (85.2%) | 125 (77.2%) | 994 (82.6%) |  |
| Y | 1,714 (16.0%) | 198 (25.3%) | 1,270 (14.8%) | 37 (22.8%) | 209 (17.4%) |  |
| Liver disease (%) |  |  |  |  |  | <0.001 |
| N | 9,272 (86.6%) | 720 (92.0%) | 7,469 (87.3%) | 146 (90.1%) | 937 (77.9%) |  |
| Y | 1,435 (13.4%) | 63 (8.0%) | 1,090 (12.7%) | 16 (9.9%) | 266 (22.1%) |  |
| ICU LOS (days) | 6 (3, 11) | 7 (3, 14) | 5 (3, 10) | 7 (3, 16) | 6 (3, 12) | <0.001 |
| ICU mortality (n (%)) |  |  |  |  |  | <0.001 |
| N | 8,409 (78.5%) | 665 (84.9%) | 6,917 (80.8%) | 111 (68.5%) | 716 (59.5%) |  |
| Y | 2,298 (21.5%) | 118 (15.1%) | 1,642 (19.2%) | 51 (31.5%) | 487 (40.5%) |  |

**Supplementary Table S2.** Multivariable COX regression analysis of different glucocorticoid treatment groups

| Characteristic | Model 1 | | | | | Model 2 | | | | | Model 3 | | | | |
| --- | --- | --- | --- | --- | --- | --- | --- | --- | --- | --- | --- | --- | --- | --- | --- |
|  | N | Event N | HR | 95% CI | p-value | N | Event N | HR | 95% CI | p-value | N | Event N | HR | 95% CI | p-value |
| Use of long-acting glucocorticoids only | 783 | 118 | — | — |  | 783 | 118 | — | — |  | 783 | 118 | — | — |  |
| Without short- or long-acting glucocorticoid use | 8,559 | 1,642 | 1.58 | 1.31, 1.91 | <0.001 | 8,559 | 1,642 | 1.51 | 1.25, 1.82 | <0.001 | 8,559 | 1,642 | 1.24 | 1.03, 1.50 | 0.024 |
| Use of both short- and long-acting glucocorticoids | 162 | 51 | 2.01 | 1.45, 2.80 | <0.001 | 162 | 51 | 2.07 | 1.49, 2.88 | <0.001 | 162 | 51 | 1.22 | 0.88, 1.71 | 0.238 |
| Use of short-acting glucocorticoids only | 1,203 | 487 | 2.92 | 2.39, 3.58 | <0.001 | 1,203 | 487 | 2.90 | 2.37, 3.55 | <0.001 | 1,203 | 487 | 1.56 | 1.26, 1.92 | <0.001 |
| P for trend |  |  |  |  | <0.001 |  |  |  |  | <0.001 |  |  |  |  | <0.001 |
| Model 1 : no covariates were adjusted  Model 2 : adjusted for Age and Gender  Model 3 : adjusted for Age, Gender, BMI, CRRT, Cancer, Liver disease, SOFA, APSIII, SAPSII, Vasopressin, HR, SBP, DBP, RR, Temperature, WBC, PCO2, PO2, Lactate, INR, Bilirubintotal, AST, Ureanitrogen, and Creatinine | | | | | | | | | | | | | | | |
