# Supplementary figures and images for: Impact of long-acting glucocorticoids on ICU mortality in septic patients with acute respiratory failure: a MIMIC-IV based cohort study
Source: Front Pharmacol. 2025 Aug 29;16:1663974. doi: 10.3389/fphar.2025.1663974 (PMC12426184; doi:10.3389/fphar.2025.1663974)

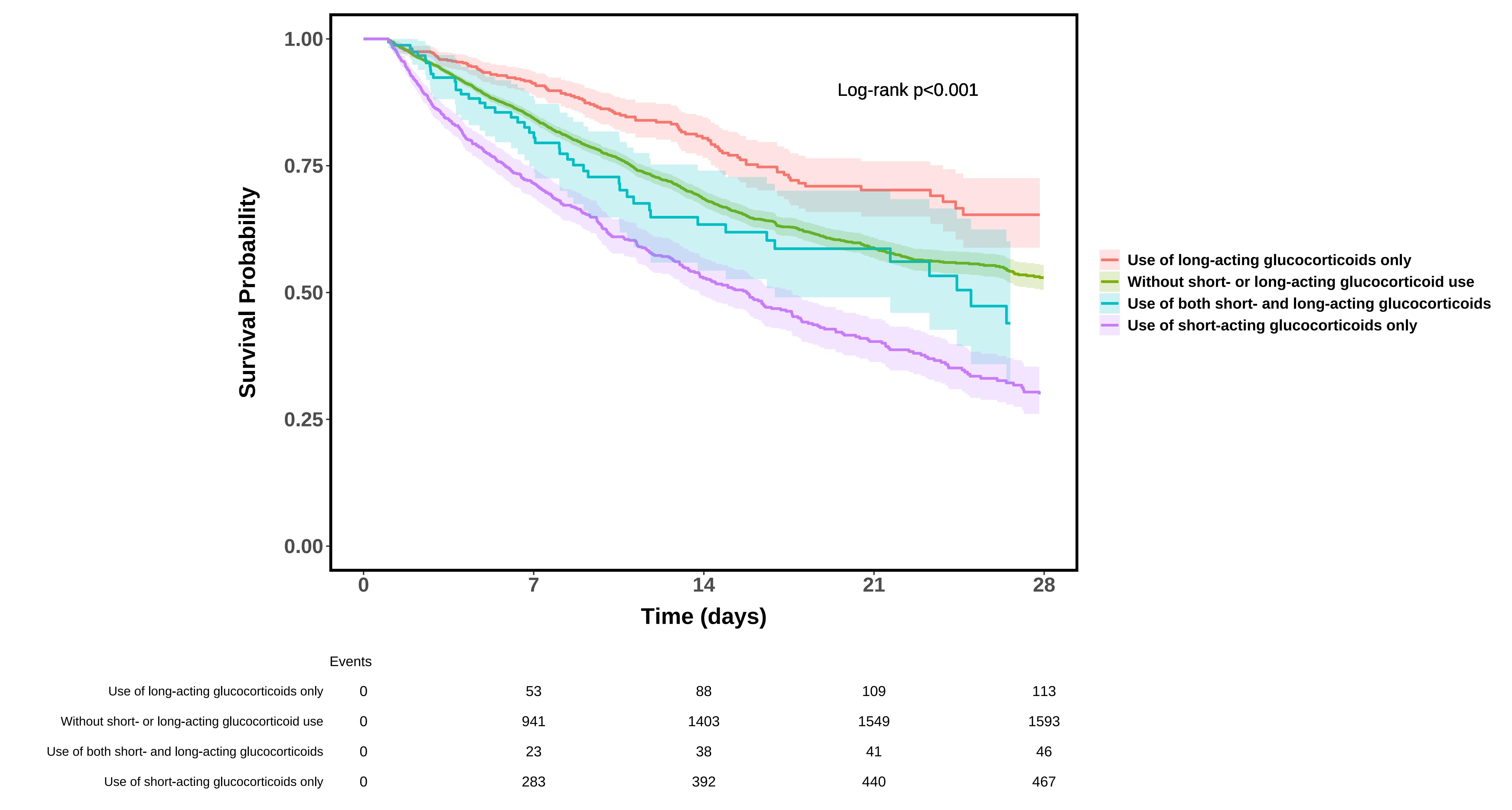

Supplement: Supplementary file 2 [file Image1.jpeg]
